# Derivation and internal validation of the multivariate toxigenic *C. difficile* diarrhea model and risk score for emergency room and hospitalized patients with diarrhea

**DOI:** 10.1017/ash.2024.58

**Published:** 2024-04-29

**Authors:** Sarah Davies, Jimmy Zhang, Yongjun Yi, Eric R. Burge, Marc Desjardins, Peter C. Austin, Carl van Walraven

**Affiliations:** 1 Department of Medicine, University of Ottawa, Ottawa, ON, Canada; 2 Institute of Health Policy, Management and Evaluation, University of Toronto, ICES, Toronto, ON, Canada; 3 Department of Epidemiology & Community Medicine, University of Ottawa, Ottawa Hospital Research Institute, ICES Ottawa, ON, Canada

## Abstract

**Background:**

Many factors have been associated with the risk of toxigenic *C. difficile* diarrhea (TCdD). This study derived and internally validated a multivariate model for estimating the risk of TCdD in patients with diarrhea using readily available clinical factors.

**Methods:**

A random sample of 3,050 symptomatic emergency department or hospitalized patients undergoing testing for toxigenic *C. difficile* at a single teaching hospital between 2014 and 2018 was created. Unformed stool samples positive for both glutamate dehydrogenase antigen by enzyme immunoassay and *tcdB* gene by polymerase chain reaction were classified as TCdD positive. The TCdD Model was created using logistic regression and was modified to the TCdD Risk Score to facilitate its use.

**Results:**

8.1% of patients were TCdD positive. TCdD risk increased with abdominal pain (adjusted odds ratio 1.3; 95% CI, 1.0–1.8), previous *C. difficile* diarrhea (2.5, 1.1–6.1), and prior antibiotic exposure, especially when sampled in the emergency department (4.2, 2.5–7.0) versus the hospital (1.7, 1.3–2.3). TCdD risk also increased when testing occurred earlier during the hospitalization encounter, when age and white cell count increased concurrently, and with decreased eosinophil count. In internal validation, the TCdD Model had moderate discrimination (optimism-corrected C-statistic 0.65, 0.62–0.68) and good calibration (optimism-corrected Integrated Calibration Index [ICI] 0.017, 0.001–0.022). Performance decreased slightly for the TCdD Risk Score (C-statistic 0.63, 0.62–0.63; ICI 0.038, 0.004–0.038).

**Conclusions:**

TCdD risk can be predicted using readily available clinical risk factors with modest accuracy.

## Introduction

*Clostridioides difficile* is the most common infectious cause of healthcare-associated diarrhea.^1^ It can impose a significant burden on the healthcare system and can cause life-threatening complications including pseudomembranous colitis, toxic megacolon, colonic perforation, sepsis, and death.^2^

Diarrhea is very common in hospitalized patients, occurring in 12%–32% of hospitalized patients with less than one-fifth caused by toxigenic *C. difficile*.^3^ It would be helpful to be able to predict the probability that a patient in the emergency room or hospital with diarrhea has toxigenic *C. difficile* diarrhea (TCdD). Patients with a high risk of TCdD might be immediately started on empiric therapy, potentially reducing their risk of developing life-threatening complications. Patients with a high risk of TCdD who test negative might have their stool analysis repeated or proceed to colonoscopy for macroscopic evidence of disease. Finally, stool analysis might be avoided for patients with a low risk of TCdD, thereby decreasing test utilization.

Published systematic reviews and meta-analyses have associated *C. difficile* infection with several demographic factors, historical data, medication exposures, physical examination findings, and laboratory results (Table 1). *C. difficile* infection risk and severity increases with increasing age.^4,5^ Patients in hospital for prolonged stays have a greater risk of hospital-acquired *C. difficile* infection.^16^ Previous TCdD was the historical factor having the strongest association with current TCdD risk.^4^ Antibiotic^4–7^ and proton pump inhibitor^4,5,10^ exposure consistently increased TCdD risk, while exposure to histamine-2 receptor antagonists^11^ and steroids^12^ has also been associated with increased risk. The only physical finding consistently associated with TCdD risk was hypotension.^15^ Finally, patients with TCdD are more likely to have leukocytosis^4^, elevated creatinine,^4,5,13^ and hypoalbuminemia.^13^ However, the independent influence that these factors have on TCdD risk is currently unknown.

**Table 1.** Summary of published studies measuring the association of factors with toxigenic *C. difficile* diarrhea (TCdD) related outcomes

					Demo		History					Medication exposure				PE	Laboratory results				
1st author	Study design	Outcome	Population	Study N	Age	Sampling day	Abdo pain	Diabetes	IBD	Heme malignant tumor	Previous cdad	Antibiotics	PPI	H2RA	Steroids	Hypotension	WBC	Eosinopenia	Creatinine	Low albumin	Lactate
**Abou Chakra** ^4^	**MA**	Any TCcD	Adults	68	↑						↑	↑	↑				↑		↑	↑	
**Deshpande** ^5^	**MA**	Recurrent TCcD	Adults	58	↑							↑	↑						↑		
**Brown** ^6^	**MA**	Communtiy-acquired TCcD	Adults	7								↑									
**Slimings** ^7^	**MA**	Hospital-acquired TCcD	Hospitalized adults	14								↑									
**Bavishi** ^8^	**SR**	Any TCdD	No restriction	27									↑								
**Leonard** ^9^	**SR**	Enteric infections	Adults	19									↑	↑							
**Trifan** ^10^	**MA**	Any TCcD	Adults	56									↑								
**Tleyjeh** ^11^	**MA**	Incident TCcD	Adults	35										↑							
**Furuya-Kanamori** ^12^	**MA**	Community-acquired TCcD	Adults	12	↑			↑	↑	↑		↑	–	–	↑				↑		
**Phatharacharukul** ^13^	**MA**	Any TCcD	Adults with CKD	20															↑		
**Bagdasarian** ^14^	**SR**	Any TCcD	Adults	116			↑									↑	↑		↑	↑	↑
**Kulaylat** ^15^	**C**	Severe TCcD	Hospitalized adults	1												↑		↑			
**Honda** ^16^	**C**	Hospital-acquired TCcD	Hospitalized adults	1		↑														↑	

Note. Up arrow (↑) indicates that variable was associated with a significantly *increased* risk of outcome. Down arrow (↓) indicates that variable was associated with a significantly *decreased* risk of outcome. Black arrow indicates that a significant association was adjusted for other factors. Gray arrows indicate that significant association was *un*adjusted. Dash (-) indicates no significant association between outcome and variable.

(CKD, chronic kidney disease; DEMO, demographics; PE, physical examination; MA, meta-analysis; SR, systematic review; C, cohort; IBD, inflammatory bowel disease; PPI, proton pump inhibitors; H2RA, histamine-2 receptor antagonist; WBC, white blood cell)

Models exist the predict the likelihood of asymptomatic toxigenic *C. difficile* carriage in the elderly,^17^ the risk that currently asymptomatic people subsequently develop TCdD,^18–20^ and the probability of ominous outcomes in TCdD patients.^21,22^ A large number of factors have been associated with *C. difficile* infection (Table 1), but no model exists that returns the probability that a patient with diarrhea has TCdD. In this study, we took a random sample of patients at our hospital from 2014 to 2018 who underwent toxigenic *C. difficile* testing to derive and internally validate a model that estimates the probability of TCdD.

## Methods

### Study setting

This study took place at the Ottawa Hospital, a 1,000-bed teaching hospital with 2 campuses that is the tertiary referral institution and trauma center for a region of approximately 1.3 million people. Annually, the Ottawa Hospital has more than 175,000 emergency department visits, 40,000 non-psychiatric admissions, and performs more than 50,000 surgical procedures. The study protocol was approved by our research ethics board and adhered to throughout (File 20210372-01H).

### Derivation of the sampling frame and sample size calculation

We created the study’s sampling frame using our hospital’s data warehouse to identify all stool analyses for toxigenic *C. difficile* conducted in our emergency department or hospital between March 15, 2014 (before which results were unavailable in our data warehouse) and December 31, 2018 (final date for which data was available when the study was initiated). Of these 29,311 specimens, we excluded those tests that were not the first sample for each patient (n = 11,608). We included only the first sample taken to be a proxy for whether patients presented with diarrhea or developed it during their admission.

From the remaining 17,703 specimens, we randomly selected 3,100 stool samples (17.5% of the final sampling frame) using the RANUNI random number function in SAS. This sample size was determined using methods presented by Riley et al.^23^ We knew that 8.7% of stool samples tested positive for toxigenic *C. difficile*, and we conservatively projected a need for 44 degrees of freedom in our model (Appendix A). We then calculated 4 required sample sizes based upon (1) a 1% error in the expected probability from the null model’s intercept (sample size = 3,060), (2) a 5% mean error around individual predictions (sample size = 1,010), (3) target shrinkage of 80% (sample size = 2,332), and (4) an optimism of 0.05 (sample size = 1,743). We took the maximum of these estimates (n = 3,060) and, anticipating an exclusion rate (due to poor documentation or unavailable records) of between 1% and 1.5%, settled on the final sample size of 3,100 patients.

### Outcome

Unformed stool samples from patients with suspected *C. difficile* infection were tested using a 2-step algorithm. Formed stool samples were rejected by the laboratory and not analyzed. Samples were initially screened for the *C. difficile*-specific glutamate dehydrogenase common antigen (GDH) by enzyme-linked immunosorbent assay (CDiffCHEK-60, TECHLAB, USA). GDH-positive samples were further tested for the presence of the tcdB gene using the Cdiff Simplexa® Universal PCR (Diasorin Molecular, USA). Patients with GDH-positive, polymerase chain reaction (PCR)-positive samples were classified with TCdD; all other patients were classified as TCdD negative.

### Data collection

To identify potential factors that we should offer to our prediction model, we first identified all covariates found to be significantly associated with any type of TCdD in published systematic reviews or meta-analyses regarding *C. difficile* diarrhea (Table 1). In addition to these 15 covariates, we identified 2 other covariates that we thought might be associated with TCdD (eosinophil count and hospitalization day of sampling) based on personal experience and cohort studies.^15,16^

We then captured values for each of these factors for all patients in our sample. We retrieved values for ten covariates (age, testing location, hospitalization antibiotic exposure, gastric acid suppression therapy, steroid exposure, and laboratory values) from our hospital data warehouse and the remaining covariates (comorbidity status, previous *C. difficile* infection, abdominal pain, systolic blood pressure) from medical record review by trained abstractors (SD, JZ, YY, EB) who were licensed and practicing physicians between June and December 2021 (Appendix A). The abstraction database interface blinded abstractors to TCdD status. Patients were classified as exposed to antibiotics *during their encounter* if they had been given any member of the beta-lactam, carbapenems, sulpha, aminoglycoside, macrolides, fluoroquinolone, tetracycline, lincosamide classes, or metronidazole prior to toxigenic *C. difficile* testing. Patients were classified as exposed to antibiotics *prior to their encounter* if they were prescribed oral or intravenous antibiotics (other than metronidazole or vancomycin) within the previous 3 months according to treating physician notes. *Steroid* exposure included prednisone, prednisolone, dexamethasone, or methylprednisolone prior to or during their encounter. We abstracted vital signs closest to and prior to toxigenic *C. difficile* testing.

### Analysis

We used logistic regression (SAS 9.4.2, Cary, NC) to measure the adjusted association of the selected variables with TCdD status. To control for the excessive influence of outliers, all continuous variables were Winsorized (ie, values below and above the 1st and 99th percentile were assigned those values, respectively).

Missing data was an issue for the laboratory tests, with the prevalence of missing values ranging from 3.5% for white blood cell count to 44.6% for serum lactate. Because these variables had skewed distributions, they were first log transformed to improve their imputation. We then used PROC MI to apply the expectation-maximization (EM) algorithm to compute the maximum likelihood estimates for imputation. All variables in the analytical data set, including outcomes, were used in the imputation model with 20 imputations.

To create the TCdD Model, we used a continuous variable transformation identification algorithm from Sauerbrei et al^24^ which combined backward elimination with an adaptive transformation identification algorithm to select the best fractional polynomial transformation for each continuous variable’s adjusted association with TCdD status using an alpha-error inclusion criterion of 0.1. Each polynomial included 2 terms and consumed 4 degrees of freedom (2 for the algorithm and 2 for the variable).^25^ Our original model-building strategy planned to offer all candidate variables (Appendix A) to the algorithm. We did not offer lactate to the model because it was missing in 44.6% of patients. In addition, we offered 2 interactions to the model: (1) an interaction between age and white blood cell count because the latter increases less in older patients^26,27^ and (2) an interaction between sampling location and previous antibiotic status because a classification tree (for an analysis not reported here) showed a strong interaction between these 2 variables. This observation might reflect that pre-admission antibiotic exposure would be more completely documented in the consultation note for patients presenting with diarrhea (and, therefore, sampled in the emergency department).

This model-building strategy was applied to each of the 20 imputation samples. The variables and transformations most commonly selected in the samples were used for the final variable selection. This model was fit in each imputation sample and final pooled parameter estimates were determined using PROC MIANALYZE.

Model performance was assessed using internal validation within 1,000 bootstrap samples. All reported performance statistics accounted for optimism using methods described by Steyerberg.^28^ We calculated the C-statistic (for discrimination) and the integrated calibration index (ICI for calibration)^29^ and used the bootstrap percentile method to calculate 95% confidence limits.^30^ C-statistics range from 0.5 to 1 with *higher* values indicating better discrimination. The ICI calculates the absolute difference between true and expected probability using a LOcal regESSion (LOESS) regression model of true values regressed on expected probabilities. ICI values range from 0 to 1 with *lower* values indicating better calibration. Finally, we used the methods described by Sullivan et al^31^ to reduce the final model to a points-based risk score, which we refer to as the TCdD Risk Score. The performance of this score was determined with the optimism-corrected C-statistic and ICI in 1,000 bootstrap samples.

## Results

Of the 3,100 randomly selected patients, 50 (1.6%) were excluded because no vital signs were recorded before the sample was taken (n = 36), no complete admission or consult note was present (n = 10), or medical record access was restricted by the patient (n = 4). This left 3,050 patients in the study cohort (Table 2). Patients had a mean age of 63.1 years (SD, 18.0), and most were admitted from the emergency room. Abdominal pain was present in almost a third of patients. Previous toxigenic *C. difficile* diarrhea (TCdD) was uncommon (1.2%). Antibiotic use *prior* to admission was recorded in a third of patients with antibiotic administration *during* the hospitalization (prior to sampling) recorded in more than half. Albumin and lactate were not measured in 22.3% and 44.6% of patients, respectively.

**Table 2.** Study cohort

Variable	Toxigenic *C. difficile* diarrhea (TCdD)		Total
	No	Yes	
	N=2,804 (91.9%)	N=246 (8.1%)	N=3,050
***Demographics***			
Mean age ± SD	63.6 ± 18.1	64.8 ± 17.4	63.7 ± 18.0
Male	1,342 (47.9%)	124 (50.4%)	1,466 (48.1%)
Admitted from emergency dept.	1,562 (55.7%)	118 (48.0%)	1,680 (55.1%)
Sampled in emergency dept.	401 (14.3%)	57 (23.2%)	458 (15.0%)
Mean hospitalization day sampling ± SD			
***History***			
Abdominal pain	794 (28.3%)	89 (36.2%)	883 (29.0%)
Diabetes mellitus	718 (25.6%)	58 (23.6%)	776 (25.4%)
Inflammatory bowel disease	150 (5.3%)	9 (3.7%)	159 (5.2%)
Hematological malignant tumor	245 (8.7%)	15 (6.1%)	260 (8.5%)
Previous CDAD	30 (1.1%)	7 (2.8%)	37 (1.2%)
***Medication exposure***			
Antibiotics: - Prior to admission	885 (31.6%)	121 (49.2%)	1,006 (33.0%)
- In hospital prior to sample	1,566 (55.8%)	120 (48.8%)	1,686 (55.3%)
Proton pump inhibitor	1,223 (43.6%)	105 (42.7%)	1,328 (43.5%)
Histamine-2 receptor antagonist	464 (16.6%)	24 (9.8%)	488 (16.0%)
Steroid	506 (18.0%)	39 (15.9%)	545 (17.9%)
***Vitals***			
Mean temperature ± SD	36.9 ± 0.8	36.9 ± 0.8	36.9 ± 0.8
Mean systolic blood pressure ± SD	124.2 ± 22.2	126.6 ± 23.2	124.4 ± 22.3
Mean heart rate ± SD	88.6 ± 18.3	90.9 ± 17.7	88.8 ± 18.2
***Mean laboratory results ±** SD **[% missing]***			
WBC × 10^9^/L	10.4 ± 8.1 [3.5]	11.2 ± 6.2 [3.6]	10.4 ± 7.9 [3.5]
Eosinophils × 10^9^/L	0.17 ± 0.35 [8.1]	0.12 ± 0.16 [11.0]	0.16 ± 0.3 [8.4]
Mean Creatinine umol/L	116.6 ± 127.1 [3.8]	137.2 ± 159.1 [4.1]	116.7 ± 127.8 [3.8]
Albumin g/L	26.8 ± 6.7 [22.4]	26.2 ± 6.1 [20.7]	26.7 ± 6.7 [22.3]
Serum Lactate, mmol/L	1.88 ± 1.34 [44.6]	1.74 ± 1.01 [56.5]	1.87 ± 1.32 [44.6]

Note. SD, standard deviation; Dept., department; CDAD, *C. difficile*-associated diarrhea; WBC, white blood cell.

TCdD was present in 246 patients (8.1%) and its status varied by several factors (Table 2). Notably, TCdD patients were notably more likely to have been exposed to antibiotics prior to admission (49.2% vs 31.6%), be sampled in the emergency department (23.2% vs 14.3%), have abdominal pain (36.2% vs 28.3%), and have previous TCdD (2.8% vs 1.1%). Mean white blood cell count and patient age were both slightly greater in those who had TCdD (11.1 × 10^9^/L vs 10.3 × 10^9^/L and 64.8 vs 63.7 years, respectively). Patients with TCdD had slightly *lower* mean eosinophil counts (0.12 × 10^9^/L vs 0.17 × 10^9^/L).

**Figure 1.**
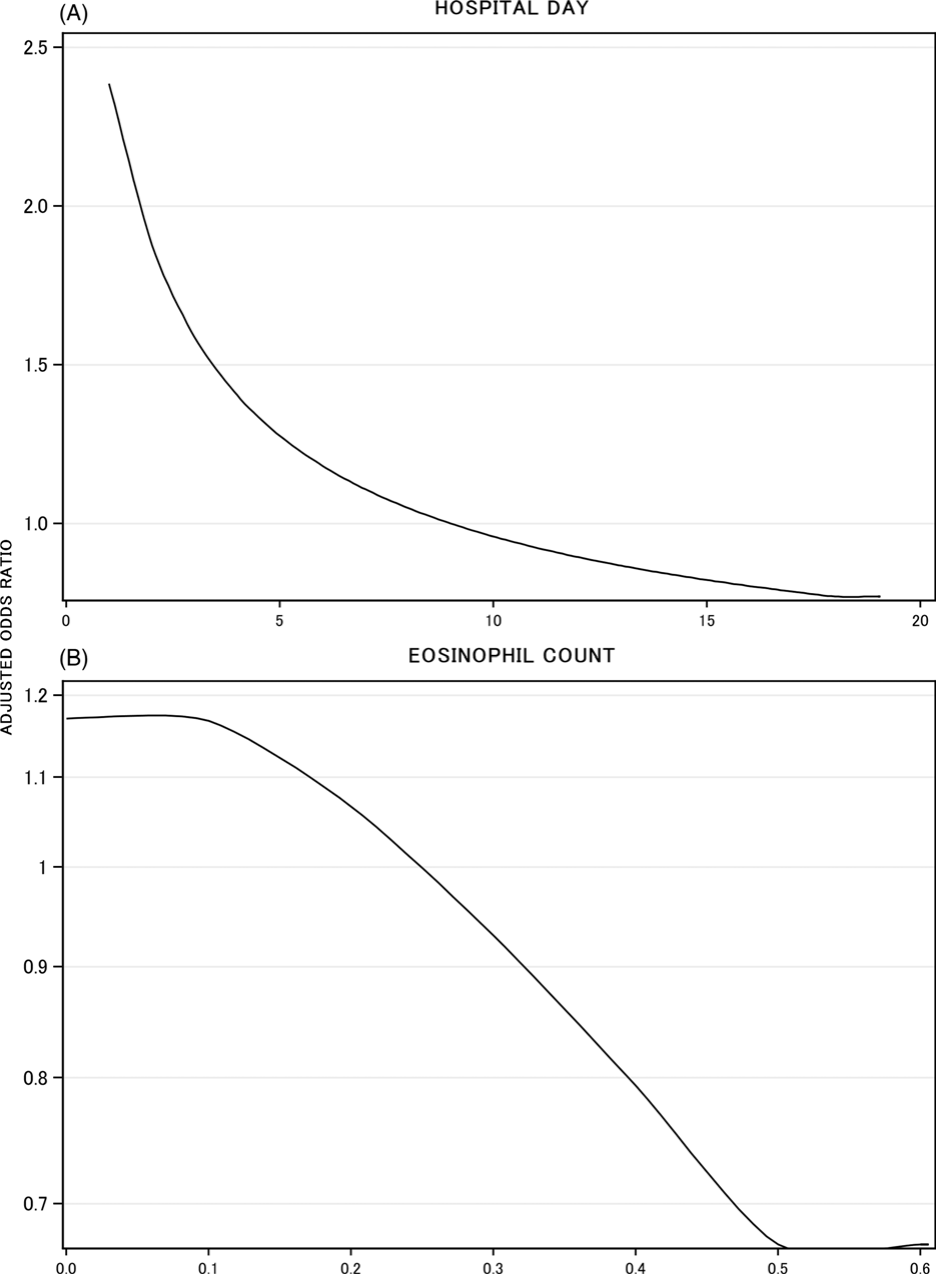
Adjusted association of hospital day and eosinophil count on toxigenic *C. difficile* diarrhea (TCdD). The adjusted association of hospital day of sample (A) and eosinophil count (B) with TCdD is presented. In both plots, the association is presented as the adjusted odds ratio (vertical axis) accounting for all other variables in the TCdD Model (Appendix B) relative to midpoint values (day 9 and eosinophil count of 0.25).

**Figure 2.**
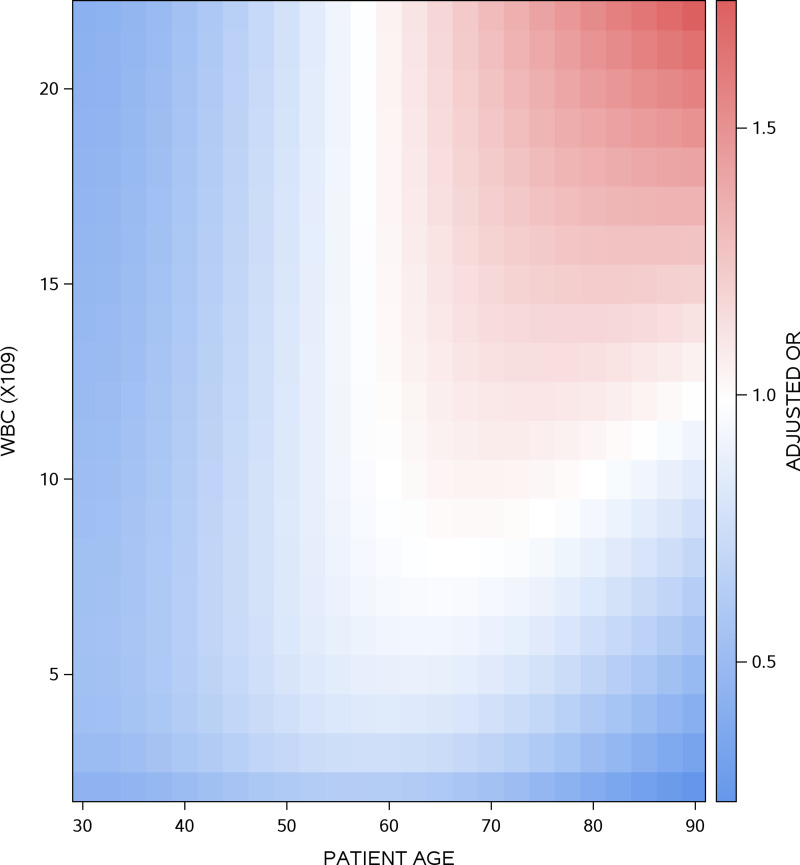
Interaction of patient age and white cell count on adjusted toxigenic *C. difficile* diarrhea (TCdD) risk. This heat map presents the adjusted likelihood of TCdD risk as a function of patient age (horizontal axis) and WBC count (left vertical axis). TCdD risk is expressed as an adjusted odds ratio (right vertical axis) relative to that of a 60-year-old patient with a WBC count of 11 (with an adjusted odds ratio of 1.0, presented as white). Adjusted odds ratios less than 1.0 (indicating *lower* TCDD risk) are blue; adjusted odds ratios exceeding 1.0 (indicating *higher* TCdD risk) are red. See Appendix B for the entire TCdD Risk Model.

The TCdD Model included 4 binary variables and 4 continuous variables along with 2 interaction terms (Appendix B). The presence of abdominal pain (adjusted odds ratio [adjOR] 1.3 [95% CI, 1.0–1.8]) and a history of previous TCdD (adjOR 2.5 [1.1–6.1]) were both independently associated with an increased risk of TCdD (Table 3). Sampling location interacted significantly with previous antibiotic exposure: increased TCdD risk associated with previous antibiotic exposure was significantly greater when patients had their stool sample taken in the emergency room (adjOR 4.2 [95% CI, 2.5, 7.0]) than when patients had their stool sample taken in hospital (adjOR 1.7 [1.3, 2.3]). In contrast, sampling location had no influence on TCdD risk in patients *without* previous antibiotic exposure. TCdD risk *de*creased with time from presentation to testing (Figure 1A) and eosinophil count (Figure 1B). Patient age and white blood cell (WBC) count interacted significantly (Appendix B). TCdD risk increased with rising WBC count predominantly when patients were older than 60 years (Figure 2). Similarly, TCdD risk increased with rising age primarily when WBC count exceeded 10 × 10^9^/L.

**Table 3.** Influence of binary variables in toxigenic *C. difficile* diarrhea (TCdD) model

Variable	Parameter estimate (standard error)	Adjusted OR (95% CI)	***P*** **-value**
Intercept	–35.86 (9.03)	–	<.0001
Abdominal pain	0.29 (0.16)	1.3 (1.0, 1.8)	.070
Previous TCdD	0.93 (0.44)	2.5 (1.1, 6.1)	.036
*Sampled in hospital*			
- No antibiotics prior to admission	REF	–	–
- Antibiotics prior to admission	0.54 (0.15)	1.7 (1.3, 2.3)	.001
*Sampled in emergency dept.*			
- No antibiotics prior to admission	–0.20 (0.29)	0.8 (0.5, 1.5)	.497
- Antibiotics prior to admission	1.43 (0.27)	4.2 (2.5, 7.0)	<.0001

Note. The influence of all binary variables in the TCdD Risk Model (Appendix B) is presented.

(OR, odds ratio; CI, confidence interval; CDAD, *C. difficile*-associated diarrhea; Dept., Department)

**Figure 3.**
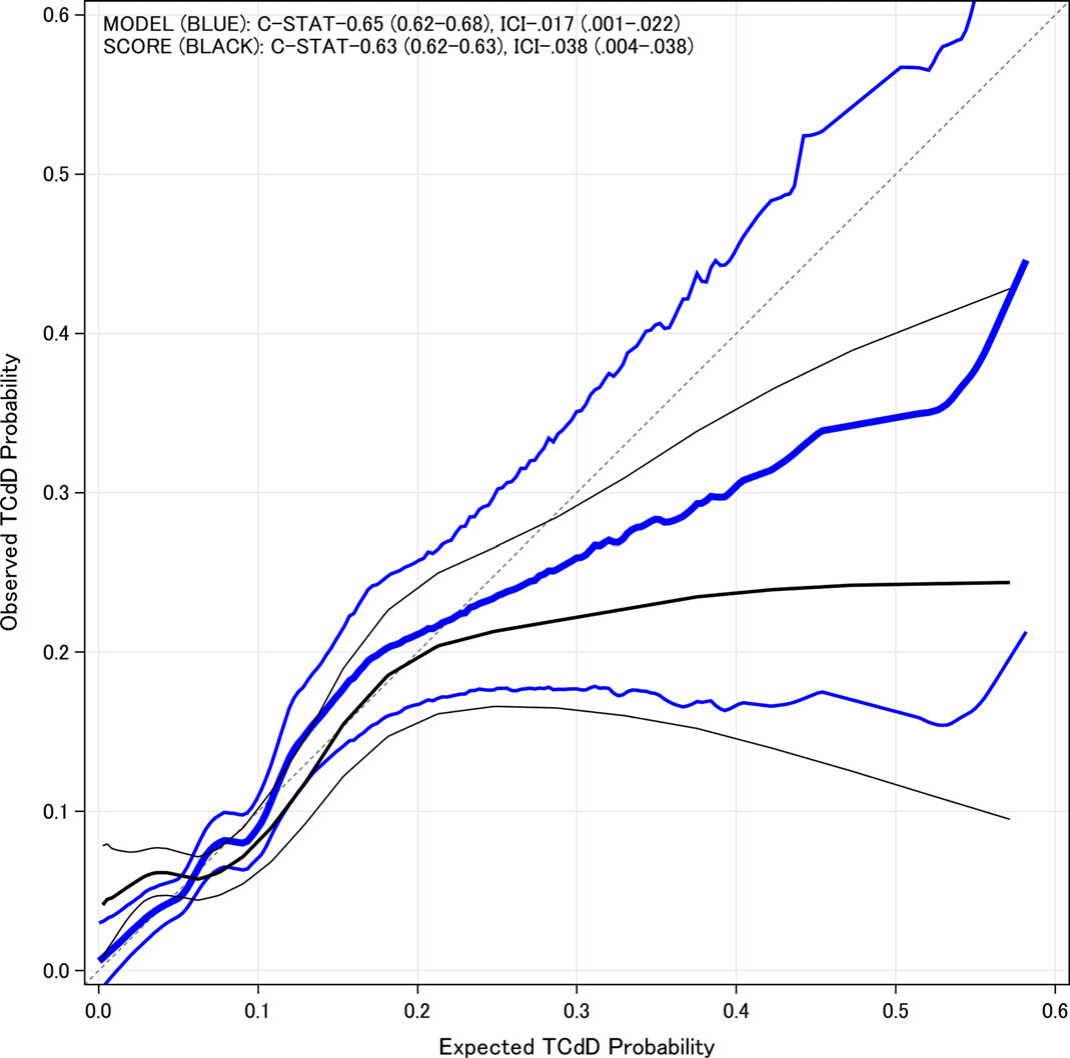
Calibration plot of toxigenic *C. difficile* diarrhea (TCdD) model and score. This figure plots the observed TCdD probability (vertical axis) against the expected TCdD probability (horizontal axis) TCdD Model (Appendix B, blue) and the TCdD probability risk score (Table 4, black). Optimism-adjusted fit statistics in the internal validation population (top left) were calculated on 1,000 bootstrap samples.

In a 1000-bootstrap internal validation, the model had an optimism-corrected C-statistic of 0.651 (95% CI, 0.621–0.676) and ICI of 0.0167 (95% CI, 0.001–0.022). Model-generated TCdD probabilities (expressed as percentages) ranged from 0.04% to 55.6% with a median value (25th–75th percentile) of 6.7% (5.0%–9.2%). Although calibration was good in patients with an expected TCdD risk of less than 20%, the model tended to *over*estimate risk when the expected risk was higher (Figure 3).

The TCdD Risk Score is presented in Table 4. A 1-point increase in the score represents the increase in TCdD risk associated with being sampled in the emergency department without previous antibiotic exposure. In our sample, the TCdD Risk Score ranged from –13 to +16, with scores that were associated with TCdD probabilities of 0.3%–52.2%, respectively (Table 4). The median risk score (25th–75th percentile) was 2 (0–4). In a 1000-bootstrap internal validation, the optimism-corrected C-statistic was 0.627 (95% CI, 0.620–0.629), and the optimism-corrected integrated calibration index was 0.038 (95% CI, 0.005–0.038) (Figure 3).

**Table 4.** Toxigenic *C. difficile* Diarrhea (TCdD) Risk Score

Factor	Points
Abdominal pain	1
Previous TCdD	5
No antibiotics pre-admission: sampled in hospital	0
Sampled in emergency dept.	–1
Antibiotics pre-admission: sampled in hospital	3
Sampled in emergency dept.	7

Eosinophil count				
≤ 0.1	0.2–0.3	0.4	0.5–0.6	> = 0.7
1	0	–1	–2	–7

Hospital day						
1	2	3–4	5–6	7–9	10–21	> 21
4	3	2	1	0	–1	–2

Points		Age (years)						
		< 35	35–<50	50–<60	60–<65	65–<70	70–<80	≥ 80
**WBC (x10** ^**9**^ **)**	**< 2**	–6	–6	–5	–5	–6	–7	–10
	**2–<5**	–3	–2	–1	–1	–2	–2	–4
	**5–<7**	–3	–2	–1	–1	–1	–1	–3
	**7–<8**	–3	–2	–1	0	0	–1	–2
	**8–<10**	–3	–2	–1	0	0	0	–1
	**10–<11**	–3	–2	–1	0	0	0	–1
	**11–<13**	–3	–2	–1	0	0	0	0
	**13–<16**	–4	–2	–1	0	1	1	1
	**16–>20**	–4	–3	–1	0	1	1	2
	**≥ 20**	–5	–3	–1	1	1	2	4

Total points	P [TCDD+] (%)	Total points	P [TCDD+] (%)	Total points	P [TCDD+] (%)
< =– 13	0.3	–3	2.4	7	15.4
–12	0.4	–2	2.9	8	18.1
–11	0.5	–1	3.5	9	21.3
–10	0.6	0	4.3	10	24.8
–9	0.7	1	5.2	11	28.7
–8	0.9	2	6.3	12	33
–7	1.1	3	7.6	13	37.5
–6	1.3	4	9.1	14	42.3
–5	1.6	5	10.9	15	47.2
–4	2	6	12.9	16+	52.2

Note. The TCdD Risk Score is calculated by adding up the points assigned to a patient’s status for abdominal pain, previous TCdD, antibiotic exposure prior to hospitalization, location of sampling, patient age, and white blood cell count. TCdD probability by risk score is provided in the bottom table. In a 1,000-bootstrap internal validation, the optimism-corrected C-statistic was 0.627 (95% CI, 0.620–0.629), and the optimism-corrected integrated calibration index was 0.0376 (95% CI, 0.005–0.038).

## Discussion

In a randomly sampled and large cohort of symptomatic patients at a single hospital, this study measured the independent association of factors previously identified to be associated with toxigenic *C. difficile* diarrhea (TCdD). We identified several factors that were independently associated to create the TCdD Model, which had moderate discrimination and good calibration. Predictive performance was slightly reduced in the TCdD Risk Score. We conclude that TCdD risk can be predicted in symptomatic patients using readily available clinical factors with modest accuracy.

The TCdD Model performed with modest accuracy despite using optimal model construction methods. First, we reviewed the literature to identify all factors significantly associated with TCdD risk in systematic reviews or meta-analyses. We then determined the status of these variables in an appropriately sized and randomly selected cohort. Therefore, we believe it is unlikely that our model’s middling predictive performance stems from missing a known important predictor or an inadequate sample size. Second, model construction used all of the techniques recommended in the Prediction model Risk Of Bias ASsessment Tool (PROBAST) criteria^32^: we used appropriate data sources and inclusion criteria; all predictors were precisely defined, were assessed without knowing outcome status, and are available to clinicians; the outcome was determined in a universal, standard fashion independent of predictor status; and we used a large patient cohort, analyzed continuous variables appropriately, appropriately accounted for missing data, included almost all randomly selected patients in our analysis, avoided predictor selection based on univariate analysis, and accounted for optimism when measuring model performance. Therefore, we don’t believe that the TCdD Model’s modest performance was due to substandard methodologies.

Several issues should be kept in mind when interpreting our results. First, although our study included a cohort that was large and randomly sampled from a valid and inclusive sampling frame, it was limited to a single institution. The validity of our model should be determined in an external population, as opposed to the interval validation with bootstrap sampling used in this study. Second, data collection was not prospective. This trait is most relevant to factors reliant upon physician documentation, such as abdominal pain and previous antibiotic use. Our analysis found that these factors were both independently associated with TCdD status (Table 3); misclassification of these important variables due to inaccurate physician documentation could mute their true association with TCdD risk. Therefore, TCdD prediction accuracy might improve with prospective data collection. Third, laboratory staff in our hospital exclude formed stools sent for *C. difficile* toxin analysis. Therefore, our study included only patients with diarrhea. However, there are 2 situations in which study patients with *C. difficile* toxin-positive stool could be misclassified with TCdD: patients colonized with *C. difficile* with diarrhea of another cause and patients with formed stool whose sample was incorrectly analyzed. We believe it to be unlikely that the prevalence of these situations was high enough to importantly alter results. Fourth, although our medical record abstractors were not told each patient’s TCdD status, they might have determined this diagnosis during abstraction which could have influenced values of abstracted covariates. Because abstractors focused on consult notes and vital signs (as opposed to microbiology results), we believe that such misclassification is unlikely to be meaningful. Fifth, “missingness” was especially common for laboratory data, especially albumin and lactate. Although we accounted for this using appropriate multiple imputation methods, model performance might improve if all patients had these tests measured, assuming that these factors are importantly predictive of TCdD status. Finally, we do not believe that our model’s performance was strong enough to be immediately impactful to clinicians—such as excluding patients for *C. difficile* toxin testing for patients with a predicted low risk of the disease. However, the predicted TCdD risk could be used by clinicians for risk stratifying patients; for example, patients with an elevated predicted TCdD risk whose initial testing is negative might undergo repeat testing or other related studies for *C. difficile* such as colonoscopy. If new TCdD predictive factors are identified in the future, our model could serve as a substrate for enhancing TCdD predictive models.

In conclusion, these data show that TCdD risk can be predicted using readily available clinical factors with modest accuracy. Further study is required to determine whether other factors improve TCdD prediction capability.

## Supporting information

Davies et al. supplementary material Davies et al. supplementary material

## Supplementary material

For supplementary material accompanying this paper visit http://doi.org/10.1017/ash.2024.58. click here to view supplementary material
