# Supplementary material for: Derivation and internal validation of the multivariate toxigenic C. difficile diarrhea model and risk score for emergency room and hospitalized patients with diarrhea
Source: Antimicrob Steward Healthc Epidemiol. 2024 Apr 29;4(1):e66. doi: 10.1017/ash.2024.58 (PMC11062795; doi:10.1017/ash.2024.58)
Supplement: Davies et al. supplementary material [file S2732494X24000585sup001.docx]

**APPENDIX A:** Summary of degrees of freedom burden and source of variables offered to models.

| **VARIABLE** | **SOURCE** | **DEGREES OF FREEDOM** |
| --- | --- | --- |
| ***MEDICATIONS*** |  |  |
| Age | DW | 4 |
| Testing location | DW | 1 |
| Hospitalization duration | DW | 4 |
| ***MEDICATIONS*** |  |  |
| - Prior antibiotic use | CHART | 1 |
| - Antibiotic exposure during encounter* | DW | 1 |
| - PPI | DW | 1 |
| - H2RA | DW | 1 |
| - Steroid | DW | 1 |
| ***LABORATORY RESULTS*** |  |  |
| - WBC | DW | 4 |
| - Serum creatinine | DW | 4 |
| - Albumin | DW | 4 |
| ***COMORBIDITIES*** |  |  |
| - Inflammatory bowel disease | CHART | 1 |
| - Hematological cancer | CHART | 1 |
| - Abdominal pain | CHART | 1 |
| - Diabetes | CHART | 1 |
| - Previous CDI | CHART | 1 |
| ***VITAL SIGNS*** |  |  |
| - Systolic Blood Pressure | CHART | 4 |
| ***INTERACTIONS*** |  |  |
| Testing location*Prior antibiotic use | CHART | 1 |
| Age*WBC | DW | 4 |

*Prior to testing.

**APPENDIX B:**  Toxigenic *C. difficile* Diarrhea (TCdD) Model

| **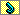Variable** | **Parameter Estimate** | **Standard Error** | **t** | **Pr > t** |  |
| --- | --- | --- | --- | --- | --- |
|  |  |  |  |  |  |
| Intercept | -35.860 | 9.025 | -3.97 | <.0001 |  |
| ***BINARY VARIABLES*** |  |  |  |  |  |
| Antibiotics Pre-Admission | 0.538 | 0.155 | 3.47 | 0.0005 |  |
| Sampled in Emergency Dept. | -0.200 | 0.294 | -0.68 | 0.4969 |  |
| Antibiotics Pre-Admission*Sampled in Emergency Dept. | 1.093 | 0.341 | 3.20 | 0.0014 |  |
| Abdominal Pain | 0.286 | 0.157 | 1.81 | 0.0698 |  |
| Previous TCdD | 0.932 | 0.443 | 2.10 | 0.0356 |  |
| ***CONTINUOUS VARIABLES*** |  |  |  |  |  |
| (Sample Day)^-0.5 | 2.232 | 0.858 | 2.60 | 0.0093 |  |
| (Sample Day)^-0.5*LN(Sample Day) | 0.844 | 0.299 | 2.82 | 0.0048 |  |
| LN(Eosinophil Count+2)^-2 | 5.511 | 3.323 | 1.66 | 0.0972 |  |
| LN(Eosinophil Count+2)^-2*LN[LN(Eosinophil Count+2)] | 6.603 | 4.399 | 1.50 | 0.1334 |  |
| (Patient Age)^(-2) | 2088.1 | 830.127 | 2.52 | 0.0119 |  |
| (Patient Age)^2 | -0.001 | 0.000251 | -2.90 | 0.0038 |  |
| [LN(WBC)+2.4]^(-2) | 0.151 | 0.051 | 2.96 | 0.003 |  |
| LN(WBC)+2.4 | -1.399 | 0.578 | -2.42 | 0.0155 |  |
| Ln(Patient Age*[Ln(WBC)+2.4]) | 5.681 | 1.986 | 2.86 | 0.0042 |  |
| (Patient Age*[Ln(WBC)+2.4])^3 | .0000000212 | .00000000874 | 2.42 | 0.0155 |  |

Values for each binary and continuous variable is multiplied by the parameter estimate. These are summed with the intercept to generate the linear predictor (lp). The expected probability of toxigenic *C. difficile* diarrhea is exp(lp)/(1 + exp(lp)).

(Dept = department; WBC = white blood cell)
